# Comparison of Clinical Characteristics and Outcomes Between MRSA and MSSA Infections Among Patients in Intensive Care Units

**DOI:** 10.3390/microorganisms13071519

**Published:** 2025-06-29

**Authors:** Gustavo Andrés Urriago-Osorio, Heiler Lozada-Ramos, Jorge Enrique Daza-Arana, Paola Andrea Ruiz-Jiménez, Giovanna Patricia Rivas-Tafurt, Diana Marcela Bonilla-Bonilla

**Affiliations:** 1 Internal Medicine Specialization Program, Department of Health, Universidad Santiago de Cali, Santiago de Cali 760035, Colombia; heiler.lozada00@usc.edu.co (H.L.-R.); paola.ruiz05@usc.edu.co (P.A.R.-J.); giovanna.rivas@clinicadeoccidente.com (G.P.R.-T.); diana.bonilla01@usc.edu.co (D.M.B.-B.); 2 Department of Research and Education, Clínica de Occidente, Santiago de Cali 760046, Colombia; 3 Genetics, Physiology, and Metabolism Research Group (GEFIME), Facultad de Salud, Universidad Santiago de Cali, Santiago de Cali 760035, Colombia; 4 Health and Movement Research Group, Facultad de Salud, Universidad Santiago de Cali, Santiago de Cali 760035, Colombia

**Keywords:** *Staphylococcus aureus*, MRSA, MSSA, mortality

## Abstract

*Staphylococcus aureus* infections are an important cause of morbidity and mortality among patients in intensive care units (ICUs), particularly those with multiple comorbidities and critical conditions. Methicillin-resistant *S. aureus* (MRSA) and methicillin-sensitive *S. aureus* (MSSA) strains differ in resistance, clinical behavior, and prognoses, making it important to understand their effects on clinical outcomes. Comparing clinical outcomes of MRSA and MSSA infections is important. This retrospective cohort study analyzed ICU patients with confirmed *S. aureus* infections at a quaternary care hospital. Demographic, clinical, and comorbidity data were collected. Poisson regression was used to analyze 7-day mortality and identify adjusted risk factors. Seven-day mortality was higher in patients with MSSA than MRSA infections, with an adjusted relative risk for MRSA of 0.380 (95% confidence interval: 0.15–0.95; *p* = 0.039). Independent risk factors for mortality included lack of an infectious disease consultation, vascular comorbidities, such as peripheral vascular disease and cerebrovascular events, chronic kidney disease, and inotropic support requirement. Patients with MRSA infections required significantly longer ventilatory support (mean 43.5 days vs. 13 days for MSSA; *p* = 0.019). *Staphylococcus aureus* infections in ICU patients were associated with poor outcomes, particularly in patients without infectious disease consultation and those with vascular comorbidities. Mortality differences between MRSA and MSSA highlight the importance of appropriate empiric therapy and standardized protocols incorporating infectious disease consultation to improve outcomes in critically ill patients.

## 1. Introduction

*Staphylococcus aureus* infections are a major cause of morbidity and mortality in hospitalized patients, particularly in those admitted to intensive care units (ICUs) due to their critical clinical condition. Among this bacterial species, methicillin-resistant *S. aureus* (MRSA) and methicillin-sensitive *S. aureus* (MSSA) are two strains with different antibiotic susceptibility, clinical behavior, and prognoses [1]. MRSA is resistant to beta-lactam antibiotics, making it a challenging pathogen to treat. Conversely, although MSSA is generally more susceptible to these antibiotics, it remains a serious threat in the ICU setting due to the vulnerability of critically ill patients [2]. Recent studies have shown an increasing trend in *S. aureus* infections in ICUs, particularly in samples from orotracheal secretions and blood cultures [3]. This trend has substantial economic implications. For example, studies conducted in Bogotá, Colombia, revealed that MRSA infections increased healthcare costs by approximately 70% compared with those of MSSA infections. This was primarily due to prolonged hospital stays, increased use of intensive care services, and the need for more expensive treatments, particularly broad-spectrum antibiotics [4].

Comparing MRSA and MSSA infections in ICUs is clinically important, as ICU patients often have immunological and physiological vulnerabilities that make them particularly prone to severe infections. ICU infections are frequently associated with prolonged hospital stays, increased use of invasive devices, and comorbidities, which complicate the treatment of bacterial infections, especially in the context of growing antibiotic resistance [5]. Therefore, understanding the differences in clinical presentation, therapeutic response, and outcomes between MRSA and MSSA infections is crucial to optimizing patient management, improving outcomes, and minimizing complications.

From an epidemiological perspective, MRSA has shown an increasing prevalence in hospital settings in recent decades, especially in ICUs, where colonization and infection rates are particularly high [6]. This increase has been associated with the intensive use of antibiotics and the presence of nosocomial-resistant strains [7]. Although common, MSSA infections differ substantially in terms of therapeutic management. Previous studies have indicated that MRSA infections tend to be associated with higher mortality rates, severe complications, and longer hospital stays than MSSA [8]. However, there is a lack of conclusive data specifically addressing these differences in ICU populations, leaving a significant gap in current knowledge.

This study aims to address the gap by comparing the clinical characteristics and outcomes of MRSA and MSSA infections in ICU patients. The findings of this study could have important implications for clinical management, guiding decisions about antibiotic use and infection prevention strategies in critically ill patients.

## 2. Materials and Methods

### 2.1. Study Design

An analytical retrospective cohort study was conducted to assess the clinical characteristics and outcomes of adult patients (aged ≥ 18 years) admitted to the ICU who developed *S. aureus* infections during their hospitalization at a quaternary-level institution in Cali, Colombia. The study period ranged from January 2015 to December 2023.

### 2.2. Study Population

The study population consisted of adult patients aged ≥18 years, who were admitted to the ICU and developed *S. aureus* infections confirmed by microbiological cultures during their hospitalization in the study period. Blood cultures and cultures of other body fluids (such as urine, sputum, and tissues) were reviewed to confirm the infections. Patients with colonization and no active infection, obstetric patients, those with more than one isolate during the same admission, and those with >10% missing data were excluded.

### 2.3. Sampling

The sample size was calculated using Pearson’s chi-square test (with or without Yates’ correction) to evaluate differences in ICU mortality between MRSA and MSSA infections. Based on data from Wang et al. [7], who reported mortality rates of 40.2% for MRSA and 23.3% for MSSA, a 95% confidence level and 80% statistical power were applied. Assuming a 1:2 exposed/nonexposed ratio and a 30% adjustment for nonresponse, the minimum required sample size was 375 patients: 125 with MRSA and 250 with MSSA. Patients were randomly selected from the institutional database using Epidat 3.1 software.

### 2.4. Comorbidities

The burden of comorbidities was assessed using the age-adjusted Charlson Comorbidity Index, with scores ranging from 0 to 37. Higher values reflected a greater burden of comorbidities.

### 2.5. Data Analysis

An exploratory analysis was initially performed to understand the relationships among variables. Independent variables were analyzed in relation to clinical outcomes, considering randomness, normal distribution, homoscedasticity, and the presence of outliers. Risk factors were described using absolute and relative frequencies for categorical data and measures of central tendency and dispersion for quantitative data. Statistically significant differences between groups (MRSA vs. MSSA) were analyzed using chi-square tests for qualitative variables, and differences between means were analyzed using Student’s *t*-test. Particular attention was paid to events of interest, such as mortality, using 95% confidence intervals (CIs). Overall and group survival were estimated using the Kaplan–Meier curve, and the log-rank test was used to compare survivals, with a significance level of <0.05 and a CI of 95%. In addition, a predictive Poisson multiple regression model was developed to evaluate the association between resistance profile and 7-day mortality. To this end, relative risks (RRs) were adjusted for the following potential confounding variables: vancomycin use, age, sex, previous hospitalization, ICU readmission, coronary artery disease, peripheral vascular disease (PVD), cerebrovascular disease (CVD), hypertension, diabetes mellitus, chronic kidney disease (CKD), chronic obstructive pulmonary disease (COPD), nutritional status, anemia, ventilatory support, inotropia, Charlson Comorbidity Index ≥5, and infectious disease consultation. Data processing and analysis were performed using STATA 16.0^®^ statistical software (StataCorp, College Station, Texas, USA).

### 2.6. Ethical Considerations

This study was approved by the Research Ethics Committee of the Clínica de Occidente, under approval number IYECDO-2198. Because of the retrospective design and the use of previously collected, anonymized data, the committee waived the requirement for informed consent in accordance with international ethical guidelines, including the Declaration of Helsinki [9]. Rigorous measures, including data anonymization and secure storage, were implemented to protect the confidentiality of participants. The study complies with current legal regulations on personal data protection according to national regulations (Article 11 of Resolution 08430 of 1993) [10].

## 3. Results

From 1 January 2015, to 31 December 2023, data were collected from medical records of 384 patients, of whom 250 had MSSA and 134 MRSA, with a prevalence of MRSA of 34.9%. Data was adjusted randomly in a 1:2 ratio such that 375 patients were analyzed: 125 in the MRSA group and 250 in the MSSA group. Regarding age, 52.8% and 48.8% of patients with MRSA and MSSA infections, respectively, were older than 65 years, and the mean age was similar in both groups. Sex was similarly distributed in both groups. The history of comorbidities was evaluated, and it was observed that coronary artery disease was present in 36.8% of patients with MRSA and in 33.6% of patients with MSSA, with no significant differences between the two groups (*p* = 0.539). The Charlson Comorbidity Index showed a trend toward being higher in patients with MRSA (5.42) compared with those with MSSA (4.94), although this difference did not reach statistical significance (*p* = 0.172). However, the percentage of patients with a Charlson index ≥5 was significantly higher in the MRSA group (63.2% vs. 52.4% in MSSA, *p* = 0.047), suggesting a higher burden of severe comorbidities in patients with MRSA infections. Several variables were identified with statistically significant differences (*p* < 0.05) between the MRSA and MSSA groups. Among the most notable findings was the mean hemoglobin level, with patients with MRSA showing a significantly lower average of 10.9 g/dL than 11.86 g/dL in the MSSA group (*p* = 0.001). In addition, a higher percentage of patients with MRSA had hemoglobin levels below 10 g/dL (36% vs. 2.8%, *p* = 0.014), potentially indicating a poorer overall condition in this group. Similarly, recent ICU admission within the past 3 months showed a significant difference, occurring in 10.4% of patients with MRSA compared with 4.8% of patients with MSSA (*p* = 0.040). Differences were also observed in infectious disease consultation, with a higher proportion of patients with MSSA not assessed by the infectious disease unit (18.4% vs. 8% in patients with MRSA, *p* = 0.008) and in the use of vancomycin, which was more frequent in the MSSA group (49.6% vs. 30%, *p* < 0.001) (Table 1)**.**

**Figure 1:**
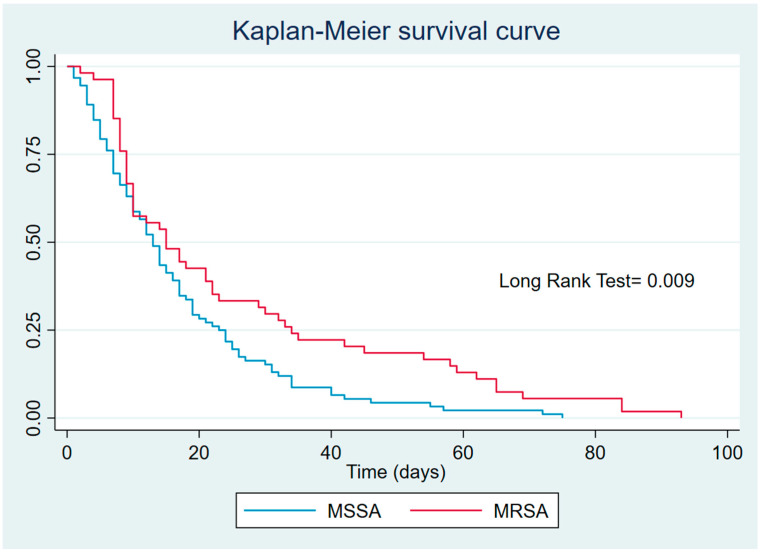
Survival analysis of patients with MRSA compared with patients with MSSA.

Survival in patients with MRSA and those with MSSA infections was assessed using a Kaplan–Meier curve based on days to death (Figure 1). Over time, cumulative survival appeared higher in patients with MRSA than those with MSSA. From the early days, the MSSA survival curve declined more rapidly than the MRSA curve, suggesting a higher risk of early mortality among MSSA-infected patients. This difference persisted over time, with mortality remaining notably higher in the MSSA group. In contrast, although MRSA patients also showed a decline in survival, the rate was slower, resulting in better overall survival throughout the observation period. The *p*-value of 0.009 indicates that the difference between the two survival curves is statistically significant, suggesting that methicillin resistance is associated with lower mortality in patients with MRSA in this context.

Clinical outcomes are presented in Table 2, showing that in-hospital mortality was 43.2% (n = 54) in patients with MRSA infections, compared with 37.2% (n = 93) in the MSSA group; this difference was not significant (*p* = 0.262). After adjusting for covariates using Poisson regression, the adjusted relative risk (aRR) was 1.180 (95% CI 0.82–1.70), indicating that there was no significant difference in the risk of in-hospital mortality between the two groups (*p* = 0.355).

Seven-day mortality trends showed mortality was lower in patients with MRSA infections (6.4%, n = 8) than in those with MSSA (11.2%, n = 28), although this difference did not reach statistical significance (*p* = 0.142). However, when a Poisson regression-adjusted analysis was performed, MRSA infections were associated with a significant reduction in the risk of 7-day mortality. The adjusted relative risk was 0.380 (95% CI: 0.15–0.95), indicating a 62% reduction in the risk of early mortality for patients with MRSA compared to those with MSSA (*p* = 0.039).

The mean time to death was significantly longer in the MRSA group, averaging 25.7 ± 23.7 days, than 16.9 ± 14.7 days in the MSSA group. The overall mean time to death in the cohort was 20.2 ± 19.0 days. The difference between the two groups was significant (*p* = 0.006), suggesting a longer duration of disease before death in patients with MSSA infections.

As for the need for acute renal replacement therapy (RRT), this was more frequent in the MRSA group, in which 38.4% of patients (n = 48) required this treatment, compared with 27.2% (n = 68) in the MSSA group. In the entire cohort, 30.9% of patients (n = 116) required RRT. This difference was significant (*p* = 0.028), suggesting higher disease severity in patients with MRSA infections. However, Poisson regression analysis yielded an aRR of 1.08 (95% CI: 0.89–1.30), with *p* = 0.415, when adjusted for confounding factors, such as the use of vancomycin.

The incidence of delirium was 30.4% (n = 38) in the MRSA group and 26.4% (n = 66) in the MSSA group. However, this difference was not significant (*p* = 0.415). The incidence of pressure ulcers was 15.2% (n = 19) in the MRSA group and 10.4% (n = 26) in the MSSA group. Although the incidence showed a trend toward being higher in the MRSA group, the difference was not significant (*p* = 0.18). In addition, 10.4% (n = 13) of patients with MRSA required reintubation, whereas 9.6% (n = 24) of patients with MSSA required reintubation. This difference was also not significant (*p* = 0.807).

Vasopressor use was high in both groups, with 76.0% (n = 95) in the MRSA group and 71.6% (n = 179) in the MSSA group. No significant difference was observed between the groups (*p* = 0.366). The mean duration of vasopressor use was 7.3 ± 7.6 days in the MRSA group and 6.4 ± 6.5 days in the MSSA group. Although the MRSA group had a longer duration, the difference was not significant (*p* = 0.331).

Inotropic use was similar in both groups, with 32.0% (n = 40) in the MRSA group and 33.6% (n = 84) in the MSSA group. No significant difference was observed between the groups (*p* = 0.756). The mean duration of inotropic support was 6.8 ± 6.8 days in the MRSA group and 3.5 ± 3.2 days in the MSSA group, showing a trend toward longer use in the MRSA group, although the difference was not significant (*p* = 0.097).

The mean duration of ventilatory support was significantly longer in the MRSA group (43.5 ± 27.2 days) than in the MSSA group (13.0 ± 12.4 days) (*p* = 0.019), suggesting greater illness severity and a prolonged need for mechanical ventilation in the MRSA group.

Patients with MRSA had a mean ICU stay of 40.4 ± 26.8 days, whereas those with MSSA had a mean ICU stay of 17.6 ± 15.9 days. Although the length of stay was longer in the MRSA group, the difference was not significant (*p* = 0.098).

In the Poisson regression analysis adjusted for 7-day mortality (Table 3), several variables were identified as statistically significant. MRSA resistance showed a significant reduction in the risk of 7-day mortality, with a relative risk ratio (RRR) of 0.39 (95% CI: 0.16–0.95; *p* = 0.039), indicating that patients with MRSA infections had a 61% lower risk of dying at 7 days than patients with MSSA infections.

Regarding comorbidities, patients with PVD had a significantly increased risk of mortality, with an RR of 2.75 (95% CI: 1.17–6.85; *p* = 0.030). Similarly, CVD was associated with an increased risk of mortality, with an RR of 2.84 (95% CI: 1.11–7.27; *p* = 0.030). Finally, CKD was also a significant predictor of increased risk of 7-day mortality, with an RR of 3.58 (95% CI: 1.14–11.19; *p* = 0.028), In addition, not being evaluated by infectious diseases was associated with a higher risk of 7-day mortality with an RR of 2.19 (95% CI: 1.05–4.54; *p* = 0.035).

## 4. Discussion

In this retrospective cohort study, we analyzed clinical outcomes in patients with MRSA and MSSA infections admitted to the ICU. We found that MRSA infections were associated with a significantly lower risk of 7-day mortality (adjusted RRR: 0.380, 95% CI: 0.15–0.950; *p* = 0.039). However, comorbidities such as PVD, CVD, and CKD were significantly associated with an increased risk of 7-day mortality. Additionally, the absence of infectious disease consultation was identified as a significant risk factor for early mortality, underscoring the importance of specialized intervention in these critically ill patients. Our study’s novelty lies in its regional scope and its focus on 7-day outcomes, a timeframe critical for ICU decision making.

The analysis of 7-day mortality revealed that MSSA infections were associated with a more rapid increase in mortality, with a 62% lower risk of early death observed in patients with MRSA infections—a finding that contrasts with those reported in previous studies. Previous studies have consistently noted that MRSA infections tend to be associated with higher mortality [11]. However, some studies have indicated that, in certain settings, MSSA infections may be associated with higher mortality than MRSA infections [12,13], which supports our findings.

For example, a retrospective cohort study reported that patients with MSSA bacteremia had higher mortality rates than those with MRSA in certain subgroups, such as individuals without severe underlying conditions or in cases where the infection progressed rapidly before appropriate antimicrobial therapy could be initiated [12]. In a study on infective endocarditis in Cali, Colombia, patients with infections caused by MSSA had a higher mortality rate than those infected by MRSA (33.3% vs. 14%, respectively) [13].

The variability in mortality outcomes between MRSA and MSSA infections may be attributed to a combination of clinical, microbiological, and methodological factors that influence study results. In addition, advances in treatment over time have contributed to a decline in MRSA-associated mortality, driven by the availability of new antimicrobials [14] and improved management of critically ill patients [15].

In contrast, vancomycin therapy has been clearly associated with increased mortality in patients with MSSA [16]. Because vancomycin is commonly used as first-line empirical treatment, it may be closely associated with the higher early mortality observed within the first 7 days. Additionally, several studies indicate that the acquisition of resistance genes in *S. aureus*, particularly in MRSA strains, may be associated with reduced virulence compared with MSSA. This phenomenon is partly attributed to the incorporation of the staphylococcal cassette chromosome mec (SCCmec), which carries the mecA gene responsible for methicillin resistance, but may also affect the expression of virulence genes. A recent study conducted in a hospital in Tunisia analyzed MRSA strains isolated from burn patients and reported that some of these strains had lower expression of key virulence factors [17]. This suggests that MRSA strains may redirect metabolic resources toward maintaining antibiotic resistance, potentially reducing their capacity to cause aggressive infections in certain clinical settings. This finding is clinically relevant, as it implies that although MRSA infections present a major challenge due to antibiotic resistance. MSSA infections may, in some cases, be more virulent or progress more rapidly, particularly when not treated promptly.

The Poisson regression analysis in our study revealed that the absence of infectious disease consultation was a significant risk factor for 7-day mortality in patients with *S. aureus* infections. These findings are consistent with previous evidence highlighting the importance of specialized follow-up in the management of severe and drug-resistant infections, particularly in the ICU setting. Early intervention by an infectious disease specialist has been shown to reduce mortality and improve clinical outcomes, primarily through the optimization of antimicrobial therapy, identification of potential sources of infection, and implementation of supportive measures that are often overlooked in less specialized care [18]. Previous studies have shown that patients who attended face-to-face infectious disease consultations had a mortality rate of 15%, compared with 23% in those without such consultation. This finding was statistically significant and was supported by a logistic regression analysis, which demonstrated an independent association between infectious disease consultation and reduced mortality (OR 0.23; 95% CI: 0.08–0.69, *p* = 0.01) [19]. Our findings, along with those of other studies, underscore that in severe infections, the absence of evaluation by an infectious disease specialist may increase the risk to patients. Infectious disease specialists often provide more accurate recommendations regarding antibiotic use and infection control measures, leading to improved initial treatment decisions.

The presence of PVD and CVD emerged in our study as independent risk factors significantly associated with increased mortality in patients with *S. aureus* infections. This is consistent with findings reported in previous studies, which have identified these comorbidities as contributors to poor prognosis in severe infections. PVD impairs blood flow, making it difficult for antibiotics to reach the infection site and weakening the local immune response, thereby worsening the infection and increasing the risk of mortality in affected patients [20]. A study in patients with *S. aureus* bacteremia reported that 30-day mortality was higher in those with vascular disease, indicating that damage to the circulatory system hinders the effectiveness of ICU treatments [21].

Patients with MRSA infections required a mean of 43.5 days of ventilatory support, significantly more than the mean 13 days observed in patients with MSSA infections (*p* = 0.019). These findings are consistent with those of previous studies, indicating that MRSA infections in the ICU are associated with prolonged ventilatory support, possibly due to increased management complexity and delayed initiation of optimal antimicrobial therapy—factors that may extend the time required to stabilize respiratory function [1]. In addition, antimicrobial resistance in MRSA infections can lead to more severe pulmonary complications, which increases the ventilatory support burden compared with infections caused by sensitive strains [22].

Among the strengths of our study is the use of a retrospective cohort design, which enabled the analysis of a large population of patients with confirmed MRSA and MSSA infections. In addition, adjustment by Poisson regression allowed us to adequately control for the major confounding variables, providing risk-adjusted estimates. However, this study has several limitations. First, the retrospective nature of the analysis limits the ability to establish causality between infections and observed outcomes, On the other hand, this prevents analyses assessing how comorbidity burden and lack of evaluation by an infectious disease specialist may influence 7-day mortality; in addition, other variables that could have influenced the results, such as the duration of antibiotic therapy, were not included. Moreover, the data were collected from a single quaternary-level institution in Cali, Colombia, which may limit the generalizability of the results to other hospital settings.

Future studies should focus on evaluating these factors in different populations and analyzing the long-term impact of MRSA and MSSA infections. Prospective studies can clarify the causal relationship between infections and clinical outcomes and the causal relationship between mortality and lack of assessment by a specialist in infectious diseases and comorbidities.

## 5. Conclusions

We found that 7-day mortality was higher in patients with MSSA infections than those with MRSA, suggesting that the rapid progression of MSSA infection, absence of infectious disease consultation, and presence of vascular comorbidities—such as PVD and CVD—are associated with increased mortality. These findings underscore the need for standardized protocols that incorporate infectious disease consultation and specific treatment strategies to reduce mortality and improve clinical outcomes in critically ill patients.

## Figures and Tables

**Table 1:** Demographic and clinical characteristics of patients.

Variable	MRSA (Exposed) n = 125		MSSA (Not Exposed) n = 250		Total n = 375		*p*-Value
	n	%	n	%	n	%	
Age							
≤65 years	59	47.2%	128	51.2%	187	49.9%	0.465
>65 years	66	52.8%	122	48.8%	188	50.1%	
Mean (±SD)	61.21	(±19.06)	62.24	(±15.98)	61.89	(±17.05)	0.583
Sex							0.510
Male	64	51.2%	137	54.8%	201	53.6%	
Female	61	48.8%	113	45.2%	174	46.4%	
Previous hospitalizations within the last 3 months	20	16.0%	29	11.6%	49	13.1%	0.233
ICU admission within the past 3 months	13	10.4%	12	4.8%	25	6.7%	0.040
Antibiotic use within the last 3 months	13	10.4%	17	6.8%	30	8.0%	0.226
Referred from another institution	66	52.8%	129	51.6%	195	52.0%	0.599
Weekend ICU admission	34	27.2%	73	29.2%	107	28.5%	0.686
Procedure upon ICU admission							
Outpatient	3	2.4%	4	1.6%	7	1.9%	0.746
Hospitalization	35	28.0%	61	24.4%	96	25.6%	
Other institution	35	28.0%	81	32.4%	116	30.9%	
Emergencies	52	41.6%	104	41.6%	156	41.6%	
ICU readmission	17	13.6%	26	10.4%	43	11.5%	
Comorbidities							
Coronary heart disease	46	36.8%	84	33.6%	130	34.7%	0.539
Peripheral vascular disease	23	18.4%	34	13.6%	57	15.2%	0.222
Cerebrovascular disease	28	22.4%	65	26.0%	93	24.8%	0.447
Diabetes mellitus	49	39.2%	81	32.4%	130	34.7%	0.192
Anemia	24	19.2%	28	11.2%	52	13.9%	0.035
COPD	18	14.4%	34	13.6%	52	13.9%	0.833
Heart failure	28	22.4%	74	29.6%	102	27.2%	0.140
Dementia	3	2.4%	12	4.8%	15	4.0%	0.264
Arterial hypertension	83	66.4%	169	67.6%	252	67.2%	0.816
Chronic kidney disease	34	27.2%	57	22.8%	91	24.3%	0.349
HIV infection	4	3.2%	1	0.4%	5	1.3%	0.026
BMI (kg/m^2^)							
<18.5	5	4.0%	10	4.0%	15	4.0%	0.362
≥30	18	14.4%	43	17.2%	61	16.3%	
18.5–24.9	52	41.6%	88	35.2%	140	37.3%	
25–29.9	28	22.4%	76	30.4%	104	27.7%	
No data	22	17.6%	33	13.2%	55	14.7%	
Charlson Comorbidity Index (CCI)							
CCI ≥ 5	79	63.2%	131	52.4%	210	56.0%	0.047
Mean (±SD)	5.42	(±3.44)	4.95	(±3.04)	5.11	(±3.18)	0.172
SOFA							
SOFA ≥ 5	35	28.0%	66	26%	101	27%	0.742
NOT assessed by the infectious disease unit	10	8.0%	46	18%	56	15%	0.008
Mean (±SD)	3.56	(±2.75)	3.23	(±2.79)	3.34	(±2.77)	0.275
Ventilatory support							
HFNC	4	3.2%	9	3.6%	13	3.5%	0.997
IMV	83	66.4%	166	66.4%	249	66.4%	
NIMV	21	16.8%	42	16.8%	63	16.8%	
ICU admission parameters							
Heart rate (bpm) (mean) (±SD)	89.48	(±22.21)	86.72	(±22.19)	87.64	(±22.21)	0.257
HR > 90 bpm	54	43.2%	91	36.4%	145	38.7%	0.202
Temperature (°C) (mean) (±SD)	36.48	(±1.05)	36.24	(±0.96)	36.32	(±1.00)	0.026
T: >38 °C or <36 °C	24	19.2%	46	18.4%	70	18.7%	0.851
Hemoglobin level (mean) (±SD) in g/dL	10.90	(±2.37)	11.86	(±2.68)	11.54	(±2.62)	0.001
Hb < 10	45	36.0%	57	22.8%	102	27.2%	0.014
Leukocyte count (mean) (±SD) in cells/µL	14,518.9	(±14,700.8)	12,601.2	(±6441.3)	13,235.3	(±9980.9)	0.081
Leu: >12,000 or <4000	75	60.0%	128	51.2%	203	54.1%	0.103
Lactate level (mmol/L) (mean) (±SD)	2.18	(±2.78)	2.22	(±2.01)	2.21	(±2.30)	0.85
Lactate > 2 mmol/L	33	26.4%	84	33.6%	117	31.2%	0.121
pH (mean) (±SD)	7.36	(±0.12)	7.37	(±0.09)	7.36	(±0.10)	0.509
pH < 7.25	10	8.0%	20	8.0%	30	8.0%	0.888
HCO_3_^−^ (mEq/L) (mean) (±SD)	20.58	(±5.70)	20.34	(±5.17)	20.42	(±5.35)	0.705
HCO_3_^−^ < 16 mEq/L	14	11.2%	32	12.8%	46	12.3%	0.625
Sodium (mEq/L) (mean) (±SD)	139.01	(±5.38)	139.57	(±5.60)	139.38	(±5.52)	0.362
Sodium > 145 or <135 mEq/L	33	26.4%	50	20.0%	83	22.1%	0.371
Potassium (mEq/L) (mean) (±SD)	4.22	(±0.75)	4.20	(±0.86)	4.21	(±0.83)	0.768
Potassium > 5 or <3.5 mEq/L	28	22.4%	68	27.2%	96	25.6%	0.544
Type of sample							
Tissue	6	4.8%	5	2.0%	11	2.9%	0.072
Peritoneal fluid	1	0.8%	1	0.4%	2	0.6%	
Blood culture	42	33.6%	71	28.4%	113	30.1%	
Bronchoalveolar lavage	2	1.6%	4	1.6%	6	1.6%	
Catheter tip	0	0.0%	1	0.4%	1	0.3%	
Orotracheal secretion	63	50.4%	154	61.6%	217	57.9%	
Urine culture	3	2.4%	0	0.0%	3	0.8%	
Skin and soft tissues	7	5.6%	14	5.6%	21	5.6%	
Cerebrospinal fluid	1	0.8%	0	0.0%	1	0.3%	
Received vancomycin	62	49.6%	75	30.0%	137	36.5%	0.000

MRSA, Methicillin-resistant *Staphylococcus aureus*; MSSA, Methicillin-sensitive *Staphylococcus aureus*; SD, Standard deviation; ICU: Intensive care unit; COPD, Chronic obstructive pulmonary disease; HIV, Human immunodeficiency virus; BMI, Body mass index; SOFA, Sequential Organ Failure Assessment; HFNC, High flow nasal cannula; IMV, Invasive mechanical ventilation; NIMV, Noninvasive mechanical ventilation; bpm, beats per minute; mEq/L, milliequivalents/liter; mmol/L, millimoles/liter.

**Table 2:** Clinical outcomes.

Variable	MRSA (Exposed) n = 125		MSSA (Not Exposed) n = 250		Total n = 375		*p*-Value
	n	%	n	%	n	%	
In-hospital mortality	54	43.20	93	37.20	147	39.20	0.262
Mortality at 7 days	8	6.4	28	11.2	36	9.6	0.142
Mean Days to death (±SD)	25.70	(±23.69)	16.91	(±14.73)	20.16	(±18.96)	0.006
Acute RRT	48	38.40	68	27.2	116	30.9	0.028
Delirium	38	30.4	66	26.4	104	27.7	0.415
Pressure ulcers	19	15.2	26	10.4	45	12.0	0.180
Reintubation	13	10.4	24	9.6	37	9.9	0.807
Vasopressor use	95	76.0	179	71.6	274	73.1	0.366
Duration of vasopressor use. days (mean) (±SD)	7.34	(±7.64)	6.49	(±6.53)	6.79	(±6.94)	0.331
Use of inotropes	40	32.0	84	33.6	124	33.1	0.756
Duration of inotrope use, days	4.98	6.81	3.46	3.26	3.95	4.73	0.097
Use of vasodilators	44	35.20	85	34.00	129	34.40	0.818
Duration of ventilatory support (mean), days (±SD)	20.51	(±43.48)	13.02	(±12.43)	15.50	(±27.17)	0.019
Length of ICU stay (mean), days (±SD)	22.48	(±40.42)	17.63	(±15.94)	19.25	(±26.76)	0.098

MRSA, Methicillin-resistant *Staphylococcus aureus*; MSSA, Methicillin-sensitive *Staphylococcus aureus*; SD, Standard deviation; ICU, Intensive care unit; RRT, Renal replacement therapy.

**Table 3:** Poisson regression in 7-day mortality.

Variable	RR	95% CI	*p*-Value
MRSA resistance	0.39	(0.16–0.95)	0.039
Not being evaluated by infectious disease specialists	2.19	(1.05–4.54)	0.035
Peripheral vascular disease	2.75	(1.17–6.85)	0.030
Cerebrovascular disease	2.84	(1.11–7.27)	0.030
Chronic kidney disease	3.58	(1.14–11.19)	0.028
Use of inotropes	3.30	(1.01–6.33)	0.047

RR: Relative Risk, MRSA, Methicillin-resistant *Staphylococcus aureus*.

## Data Availability

The data presented in this study are available on request from the corresponding author. The data are not publicly available due to privacy.

## Abbreviations

The following abbreviations are used in this manuscript:

BMI	Body mass index
CI	Confidence interval
CKD	Chronic kidney disease
COPD	Chronic obstructive pulmonary disease
CVD	Cerebrovascular disease
DM	Diabetes mellitus
HIV	Human immunodeficiency virus
HFNC	High flow nasal cannula
ICU	Intensive care unit
IMV	Invasive mechanical ventilation
MRSA	Methicillin-resistant *Staphylococcus aureus*
MSSA	Methicillin-sensitive *Staphylococcus aureus*
NIMV	Noninvasive mechanical ventilation
PVD	Peripheral vascular disease
RR	Relative risks
RRR	Relative risk ratio
RRT	Renal replacement therapy
SOFA	Sequential Organ Failure Assessment
